# Self-Efficacy as a Mediator Between Caregiver Burden, Health Literacy, and Contribution to Self-Care in Inflammatory Bowel Disease

**DOI:** 10.1007/s10620-025-09577-9

**Published:** 2025-12-03

**Authors:** Daniele Napolitano, Mattia Bozzetti, Francesco Petrosino, Silvia Cilluffo, Francesca Trotta, Gianluca Pucciarelli, Davide Bartoli, Alessio Lo Cascio, Ercole Vellone

**Affiliations:** 1 CEMAD–Fondazione Policlinico Gemelli IRCCS, Rome, Italy; 2 Direction of Health Professions, ASST Cremona, Cremona, Italy; 3 Direction of Health Professions, ASL Salerno, Salerno, Italy; 4 Department of Biomedical Sciences for Health, University of Milan, Milan, Italy; 5 AOU Sant’Andrea Hospital, Rome, Italy; 6 Department of Biomedicine and Prevention, Tor Vergata University, Rome, Italy; 7 Interdisciplinary Department of Wellbeing, Health and Environmental Sustainability - BeSSA Department, Sapienza University of Rome, 02100 Rieti, Italy; 8 La Maddalena Cancer Center, Via San Lorenzo 312, Palermo, Italy; 9 Department of Nursing and Obstetrics, Wroclaw Medical University, Wrocław, Poland

**Keywords:** Caregiver burden, Self-Care, Self-Efficacy, Inflammatory Bowel Disease, Health literacy, Structural equation modeling

## Abstract

**Purpose:**

Informal caregivers play a critical role in supporting Self-Care behaviors in patients with inflammatory bowel disease (IBD). However, the extent to which caregiver burden, health literacy, and Self-Efficacy predict their contribution to patient Self-Care remains unclear, and the potential mediating role of Self-Efficacy has not yet been established.

**Methods:**

A multicenter cross-sectional study was conducted across nine Italian IBD centers. Caregivers completed validated instruments assessing burden (Zarit Burden Interview, score range 0–88; higher scores indicate greater burden), health literacy (Single Item Literacy Screener, 1–5; scores > 2 indicate inadequate literacy), Self-Efficacy (CSE-CSC, standardized 0–100; higher scores indicate greater confidence), and caregiver contribution to Self-Care (CC-SC-CII, standardized 0–100 across maintenance, monitoring, and management; higher scores indicate greater contribution). Structural equation modelling explored direct and indirect associations among study variables.

**Results:**

Among 275 caregivers, the mean age was 51 years (*SD* = 13); 160 (58%) were female and 115 (42%) male. Mean contribution scores were 54.99 (*SD* = 25.62) for Self-Care Maintenance, 67.33 (*SD* = 32.28) for Self-Care Monitoring, and 56.18 (*SD* = 23.15) for Self-Care Management. Mean Self-Efficacy was 72.41 (*SD* = 18.42). Self-Efficacy significantly predicted Self-Care Monitoring  (*β* = 0.34, *p* < 0.001) and Self-Care Management (*β* = 0.42, *p* < 0.001), while burden showed modest but significant associations across domains. Health literacy demonstrated no significant effects, and no mediation pathways were identified.

**Conclusion:**

Self-Efficacy emerged as a central determinant of caregiver contribution to patient Self-Care in IBD, particularly for Self-Care Monitoring  and Self-Care Management  behaviors. Interventions aimed at strengthening caregiver confidence and tailored to gender- and disease-specific needs may enhance the quality of caregiving and ultimately support better patient outcomes.

**Supplementary Information:**

The online version contains supplementary material available at 10.1007/s10620-025-09577-9.

## Introduction

Inflammatory Bowel Diseases (IBD), including Crohn’s disease (CD) and ulcerative colitis (UC), are chronic, relapsing conditions that affect both patients and their informal caregivers [1, 2]. The fluctuating symptoms—such as abdominal pain, fatigue, diarrhoea, and psychological distress—can reduce patients’ quality of life and heighten their dependence on caregivers, particularly during flare-ups [3–5].

Informal caregivers play a crucial role in the daily management of chronic illness, yet their contribution often comes at a cost. In IBD, caregivers frequently experience high levels of stress, anxiety, and emotional burden, which may impair their ability to provide effective support [6–9].

Such strain can compromise symptom monitoring, adherence encouragement, and communication with healthcare providers, ultimately affecting patient outcomes and increasing healthcare utilization [10–12]. Beyond burden, caregivers contribute actively to patient Self-Care, defined as the ability to maintain health, monitor symptoms, and manage illness. According to the Middle-Range Theory of Self-Care in Chronic Illness [13]. Self-Care comprises three processes: Maintenance (promoting physical and emotional stability), Monitoring (detecting changes in health status), and Management (responding effectively to symptoms). In IBD, caregivers may support medication adherence, identify early signs of relapse, and facilitate timely contact with healthcare professionals [14–16]. Despite this central role, limited research has explored how caregiver characteristics influence their contribution to patient Self-Care. Most studies have focused on patient-related factors such as Self-Care Management  or psychological attributes [17, 18], overlooking the dyadic nature of IBD care, where patients and caregivers collaboratively manage treatment and symptoms [19]. Within this framework, caregiver burden and health literacy have emerged as potential determinants of caregiving effectiveness.

Caregiver burden—encompassing emotional, physical, and logistical strain—may reduce motivation and engagement, while low health literacy can hinder understanding of medical information and decision-making [19–21]. These factors may operate both directly and indirectly through Self-Efficacy, a mediator identified by the Middle-Range Theory of Self-Care that links knowledge, stress, and behavioral outcomes [6, 22]. However, the combined or independent effects of burden and health literacy on caregiver contribution to IBD Self-Care remain unclear.

## Methods

### Aim

The study aimed to examine the associations among caregiver burden, health literacy, and caregivers’ contributions to Self-Care, and to test whether Self-Efficacy mediated these relationships.

### Study Design and Setting

A multicentre cross-sectional study was conducted between April and June 2024 in nine Italian IBD centres, following the Strengthening the Reporting of Observational Studies in Epidemiology (STROBE) guideline [23].

### Participants

Primary informal caregivers of adult patients with IBD were recruited. Inclusion criteria were age ≥ 18 years, involvement in caregiving for at least six months, and care recipients with a confirmed IBD diagnosis for ≥ 12 months. Caregivers of patients with major comorbid chronic conditions were excluded to ensure sample homogeneity.

### Measures

Data were collected through a self-administered questionnaire that included sociodemographic variables (age, sex, employment status) and clinical variables (type of IBD).

### Caregiver Contribution to Self-Care

Caregiver contribution to patient Self-Care was assessed with the 19-item Caregiver Contribution to Self-Care of Chronic Illness Inventory (CC-SC-CII) [21]. Items are scored on a 5-point Likert scale (1 = never, 5 = always). The scores for the three domains (Maintenance, Monitoring, and Management) are standardised on a scale of 0 to 100, with higher scores reflecting better Self-Care. A score of 70 or above indicates “adequate” Self-Care.

### Caregiver Burden

Caregiver burden was measured using the 22-item Zarit Burden Interview (ZBI) [24, 25]. Each item is rated on a 5-point scale (0 = never, 4 = nearly always). The total score ranges from 0 to 88, with higher scores reflecting greater perceived burden.

### Health Literacy

Health literacy was assessed with the Single Item Literacy Screener (SILS) [26], scored on a 5-point scale (1 = never, 5 = always) in response to difficulty in reading health-related material. Scores > 2 indicate inadequate functional health literacy, whereas lower scores indicate adequate reading ability.

### Caregiver Contribution to Self-Efficacy

Caregiver Self-Efficacy was measured using the 10-item Caregiver Self-Efficacy in Contributing to Patient Self-Care Scale (CSE-CSC) [27, 28]. Items are scored on a 5-point scale (1 = not confident at all, 5 = very confident). Raw scores are standardised to a 0–100 scale, with higher scores reflecting greater confidence in supporting patient Self-Care across Maintenance, Monitoring, and Management activities.

All instruments have been previously validated with strong psychometric properties in international and Italian samples. No additional validation analyses were performed in this study.

### Statistical Analysis

Descriptive statistics were computed for all variables, expressed as mean (SD) for continuous variables and *n* (%) for categorical variables. Structural equation modelling (SEM) was conducted using the lavaan package in R (version 4.5.0) with robust maximum likelihood (MLR) estimation to test hypothesised direct and indirect relationships among caregiver burden, health literacy, Self-Efficacy, and Self-Care contribution domains (Maintenance, Monitoring, Management).

Structural equation modeling (SEM) was employed to evaluate the hypothesized associations among perceived burden, Self-Efficacy, and the three domains of caregiver contribution to Self-Care: Maintenance, Monitoring, and Management. Separate SEMs were tested for each Self-Care contribution dimension using the lavaan package in *R* 4.5.0, with robust maximum likelihood (MLR) estimation to account for potential deviations from normality. All models included age, gender, and occupational status (coded as worker vs. non-worker) allowing for the control of potential demographic confounding in the estimation of direct and indirect paths.

To optimize model stability and reduce the number of estimated parameters, item parceling guided by results from preliminary confirmatory factor analyses was applied to the latent constructs. Parcels were created using the item-to-construct balance method (ICB), also known as domain-representative parceling [29, 30], and can be seen in Supplementary File 1.

All structural paths, including indirect effects, were estimated using bootstrapped standard errors and bias-corrected confidence intervals based on 1000 resamples. This approach provided robust inferences for the mediation effects tested in each model. All inferential conclusions are drawn exclusively from the SEM to avoid Type *I* error inflation.

## Results

A total of 275 caregivers participated in the study. To provide a basic cohort picture (Supplementary File 2), we present descriptive subgroup distributions (diagnosis, caregiver gender, caregiver occupation, and patient age) that contribute to Self-Care Domains. Complete descriptive statistics are reported in Table 1.

**Table 1:** Sociodemographic and clinical characteristics of caregivers and descriptive statistics of study instruments (n = 275).

Variable	Value
Age, M (SD)	51 (13.29)
Gender, n (%)	Female: 160 (58.2%)
	Male: 115 (41.8%)
Worker status, n (%)	Worker: 184 (66.9%)
	Non-worker: 91 (33.1%)
Pathology	CD 131 (47.6%)
	UC 144 (52.4%)
Caregiver contribution to Self-Care Maintenance, M (SD)	54.99 (25.62)
Caregiver contribution to Self-Care Monitoring, M (SD)	67.33 (32.28)
Caregiver contribution to Self-Care Management, M (SD)	56.18 (23.15)
Caregiver burden, M (SD)	14.54 (11.55)
Health literacy, M (SD)	1.8 (0.97)
Caregiver Self-Efficacy in contributing to patient Self-Care, M (SD)	72.41 (18.42)

*CD* Crohn’s disease, *UC* Ulcerative colitis, *SD* Standard deviation

### Caregiver Contribution to Self-Care—Maintenance

The SEM for Caregiver Contribution to Self-Care Maintenance showed good model fit, *χ*^*2*^(75) = 120.17, *p* = 0.001, RMSEA = 0.047, 90% CI [0.030, 0.062], SRMR = 0.054, CFI = 0.972, TLI = 0.965. Caregiver burden showed a small positive association with Caregiver Contribution to Self-Care Maintenance (*β*≈0.13, *p* = 0.042). Caring for *CD* was associated with higher caregiver contribution to Maintenance (*β*≈ = −0.18, *p* = 0.010).

Self-Efficacy, health literacy, age, sex, and employment were not significant. No indirect effects via Caregiver Self-Efficacy in Contributing to Patient Self-Care were detected. The model explained 2.5% of the variance in Caregiver Self-Efficacy in Contributing to Patient Self-Care and 7.0% of the variance in Caregiver Contribution to Self-Care Maintenance (Table 2).

**Table 2:** Direct, indirect, and total effects of caregiver burden, health literacy, and Self-Efficacy on caregiver contribution to Self-Care domains (Maintenance, Monitoring, and Management) derived from structural equation modelling.

Effect	Caregiver contribution to Self-Care Maintenance estimate [95%IC]	Caregiver contribution to Self-Care Monitoring estimate [95%IC]	Caregiver contribution to Self-Care Management estimate [95%IC]
Direct effects			
Caregiver burden → Caregiver contribution to Self-Care	**0.13 [–0.01, 0.30]**	**0.13 [–0.01, 0.30]**	**0.13 [–0.01, 0.30]**
Caregiver burden → Caregiver Self-Efficacy in contributing to patient Self-Care	–0.13 [–0.34, 0.07]	–0.13 [–0.34, 0.07]	–0.13 [–0.34, 0.07]
Health literacy → Caregiver Self-Efficacy in contributing to patient Self-Care	–0.07 [–0.17, 0.02]	–0.07 [–0.17, 0.02]	–0.07 [–0.17, 0.02]
Self-Efficacy caregiver → Caregiver contribution to Self-Care	0.11 [–0.03, 0.23]	**0.11 [–0.03, 0.23]**	**0.11 [–0.03, 0.23]**
Health literacy → Caregiver contribution to Self-Care	–0.07 [–0.17, 0.02]	–0.07 [–0.17, 0.02]	–0.07 [–0.17, 0.02]
Age → Caregiver contribution to Self-Care	–0.00 [–0.01, 0.01]	**–0.00 [–0.01, 0.01]**	–0.00 [–0.01, 0.01]
Gender (0 = Female; 1 = Male) → Caregiver contribution to Self-Care	0.03 [–0.17, 0.22]	0.03 [–0.17, 0.22]	**0.03 [–0.17, 0.22]**
Employment (Worker) → Caregiver contribution to Self-Care	–0.14 [–0.38, 0.09]	–0.14 [–0.38, 0.09]	–0.14 [–0.38, 0.09]
Pathology → Caregiver Self-Efficacy in contributing to patient Self-Care	–0.09 [–0.28, 0.09]	–0.09 [–0.28, 0.09]	–0.09 [–0.28, 0.09]
Pathology (0 = *CD*; 1 = *UC*) → Caregiver contribution to Self-Care	**–0.26 [–0.46, –0.07]**	–0.26 [–0.46, –0.07]	–0.26 [–0.46, –0.07]
Indirect effects			
Caregiver burden → Caregiver Self-Efficacy in contributing to patient Self-Care → Caregiver contribution to Self-Care	–0.01 [–0.05, 0.01]	–0.01 [–0.05, 0.01]	–0.01 [–0.05, 0.01]
Health literacy → Caregiver Self-Efficacy in contributing to patient self-care → Caregiver contribution to Self-Care	–0.01 [–0.03, 0.00]	–0.01 [–0.03, 0.00]	–0.01 [–0.03, 0.00]
Pathology → Caregiver Self-Efficacy in contributing to patient Self-Care → Caregiver contribution to Self-Care	–0.01 [–0.04, 0.01]	–0.05 [–0.17, 0.04]	–0.04 [–0.12, 0.04]

Bold values are statistically significant paths

*CD* Crohn’s disease; *UC* Ulcerative colitis; 95%*CI* 95% Confidence interval

### Caregiver Contribution to Self-Care—Monitoring

The SEM for Caregiver Contribution to Self-Care Monitoring demonstrated acceptable model fit, *χ*^*2*^(75) = 137.19, *p* < 0.001, RMSEA = 0.055, 90% *CI* [0.040, 0.069], SRMR = 0.054, CFI = 0.970, TLI = 0.962. Caregiver Contribution to Self-Care monitoring was significantly predicted by Caregiver Self-Efficacy in Contributing to Patient Self-Care (*β* = 0.34, *p* < 0.001) and Caregiver Burden (β = 0.30, p < 0.001). Health literacy and demographic covariates were not significant. No indirect effects via self-efficacy emerged. The model explained 2.6% of the variance in Self-Efficacy and 20.5% in Caregiver Contribution to Self-Care Monitoring (Table 2).

### Caregiver Contribution to Self-Care—Management

The SEM for Caregiver Contribution to Self-Care Management showed good model fit, χ^2^(75) = 133.490, *p* < 0.001, RMSEA = 0.053, 90% *CI* [0.038, 0.068], SRMR = 0.058, CFI = 0.959, TLI = 0.949. Caregiver Contribution to Self-Care for Management was significantly predicted by Caregiver Self-Efficacy in Contributing to Patient Self-Care (*β* = 0.42, *p* < 0.001), Caregiver Burden (*β* = 0.17, *p *= 0.019), and Male caregivers reported lower contribution to Caregiver Contribution to Self-Care Management (*β* = –0.19, *p* = 0.009). Health literacy, age, employment, and diagnosis were not significant. No indirect effects via Self-Efficacy were observed. The model explained 2.5% of the variance in Self-Efficacy and 25.8% of the variance in Caregiver Contribution to Self-Care Management (Table 2).

## Discussion

This study provided new insights into how caregivers of patients with IBD contribute to their patients’ Self-Care and how their own burden, health literacy, and Self-Efficacy influence these contributions. Self-Efficacy emerged as the strongest direct predictor of caregiver engagement in symptom Self-Care Monitoring and Self-Care Management, whereas health literacy showed a minimal role. Burden displayed small but significant associations, suggesting that emotional strain does not necessarily lead to reduced caregiving involvement.

The association between burden and caregiving contributions aligns with previous findings in IBD and other chronic illnesses [9, 10, 31]. Even caregivers experiencing moderate burden, as measured by the ZBI, remained active contributors to patient Self-Care. This may indicate a compensatory process known as “adaptive engagement,” in which caregivers maintain high vigilance and reactivity despite emotional fatigue [32].

This activation appeared domain-specific, with burden affecting Self-Care Monitoring and Management, but not Self-Care Maintenance, consistent with evidence that stress influences reactive behaviors more than routine ones [8, 33]. Qualitative research has shown that caregivers of patients with CD often experience helplessness and fear of doing “too little,” reflecting the unpredictability and perceived severity of the disease [31]. In our study, the CD cohort elicited higher caregiver contributions in Self-Care Maintenance, likely due to the perceived severity and instability of the condition.

Caregiver Self-Efficacy was the most consistent predictor of engagement across domains, echoing findings from cardiovascular research [16], where Self-Efficacy fosters proactive coping and buffers stress. Because it is modifiable, Self-Efficacy represents a key clinical target. Evidence indicates that brief interventions—such as psychoeducation, role-playing, or digital coaching—can enhance caregiving confidence and competence [19]. Contrary to expectations derived from the Middle-Range Theory of Self-Care of Chronic Illness, SEM did not confirm a mediating effect of Self-Efficacy between burden or health literacy and caregiving behaviors. This suggests that Self-Efficacy may interact with other psychological constructs, such as resilience, dyadic coping, or perceived reciprocity [22], which were not assessed in this study.

In line with these observations, all three SEM models demonstrated an excellent global fit (CFI > 0.95; RMSEA < 0.06; SRMR < 0.06), indicating that the hypothesized measurement models were both statistically robust and theoretically coherent. Nevertheless, the amount of variance explained in the outcomes varied: the models accounted for a modest portion of the variance in Self-Care Maintenance (*r*^*2*^ = 7.0%), but considerably more in Self-Care Monitoring (20.5%) and Self-Care Management (25.8%), suggesting that these domains are more sensitive to psychological and contextual factors.

Contrary to studies in other chronic illnesses [11, 18], health literacy, assessed by the SILS, was not associated with Self-Efficacy or contribution. This may be due to the SILS capturing only functional literacy rather than interactive or critical components. Broader instruments, such as the Health Literacy Questionnaire (HLQ) or the HLS-EU-Q, could better represent caregivers’ abilities to process and apply health information [34, 35]. Another explanation is that caregivers in highly specialized IBD centers benefit from continuous professional guidance, reducing the impact of literacy on caregiving behaviors [36].

Differences by disease and gender were evident. Caregivers of patients with CD reported higher engagement in Self-Care Maintenance, consistent with the perception of CD as more severe and unpredictable than UC. Male caregivers reported lower contributions in Self-Care Management, in line with studies showing lower emotional engagement and greater avoidance among men [31]. These findings underscore the need for gender-sensitive and condition-specific interventions to improve emotional coping and caregiving competence.

Sociocultural factors also influence caregiver burden and engagement. Prior studies demonstrate that caregivers’ experiences vary widely across regions, shaped by healthcare accessibility, stigma, and social expectations [31, 33, 37].

Recent large-scale studies have highlighted that caregiving in IBD is deeply influenced by cultural and healthcare contexts [37, 38]. For instance, in France, approximately half of caregivers reported significant professional or sexual life impairments related to caregiving demands [38]. Similarly, in several Asian countries, around 65% of caregivers experienced moderate-to-severe burden, compounded by limited healthcare access and pervasive stigma [37]. Findings from Italian IBD-specialised units suggest that public healthcare coverage and multidisciplinary care models may partially buffer these effects [39–41]. Nonetheless, structured psychological support, formal caregiver training, and digital resources remain inconsistently available. Such gaps contrast with international recommendations, which emphasize equipping informal caregivers with psychosocial skills and disease-specific knowledge to ensure sustainable care delivery [42].

These findings have several practical implications. Systematic assessment of caregiver burden and Self-Efficacy should become a routine part of IBD management, particularly during disease flares when caregiving demands intensify [43, 44]. Early identification of high-risk caregivers may prevent negative outcomes for both patients and caregivers. Moreover, tailored and culturally sensitive interventions are needed for vulnerable subgroups, including male caregivers, those with low health literacy, and parents of young adults with IBD.

Digital and e-health platforms also represent a promising avenue to provide remote education, enhance Self-Efficacy, and improve access to professional and peer support [45, 46]. Finally, developing sustainable caregiver support pathways requires a coordinated, interdisciplinary approach that integrates nurses, psychologists, and social workers [39]. These collaborative models can both reduce caregiver strain and empower caregivers as active partners in patient care.

This study’s strengths include the multicentre design, use of validated instruments, and application of SEM to examine complex relationships. Limitations include the cross-sectional design, reliance on self-report measures, and the use of a single-item health literacy screener, which may not capture multidimensional literacy. Future longitudinal and mixed-methods research should evaluate how caregiver burden and contributions evolve over time and test interventions aimed at strengthening Self-Efficacy and resilience.

In conclusion, Caregiver Self-Efficacy in contributing to patients’ Self-Care remains a pivotal determinant of support in IBD Self-Care, especially in Self-Care Monitoring  and Self-Care Management. Enhancing caregiver confidence through education, peer mentorship, and digital tools could meaningfully improve outcomes for both caregivers and patients.

## Supplementary Information

Below is the link to the electronic supplementary material.

Supplementary file1 (DOCX 102 KB)

Supplementary file2 (DOCX 102 KB)

## Data Availability

The dataset generated and analyzed during the current study is available from the corresponding author on reasonable request.
