# Correction: Self-Efficacy as a Mediator Between Caregiver Burden, Health Literacy, and Contribution to Self-Care in Inflammatory Bowel Disease

**DOI:** 10.1007/s10620-026-09942-2

**Published:** 2026-05-08

**Authors:** Daniele Napolitano, Mattia Bozzetti, Francesco Petrosino, Silvia Cilluffo, Francesca Trotta, Gianluca Pucciarelli, Davide Bartoli, Alessio Lo Cascio, Ercole Vellone

**Affiliations:** 1 CEMAD–Fondazione Policlinico Gemelli IRCCS, Rome, Italy; 2 Direction of Health Professions, ASST Cremona, Cremona, Italy; 3 Direction of Health Professions, ASL Salerno, Salerno, Italy; 4 Department of Biomedical Sciences for Health, University of Milan, Milan, Italy; 5 AOU Sant’Andrea Hospital, Rome, Italy; 6 Department of Biomedicine and Prevention, Tor Vergata University, Rome, Italy; 7 Interdisciplinary Department of Wellbeing, Health and Environmental Sustainability - BeSSA Department, Sapienza University of Rome, 02100 Rieti, Italy; 8 La Maddalena Cancer Center, Via San Lorenzo 312, Palermo, Italy; 9 Department of Nursing and Obstetrics, Wroclaw Medical University, Wrocław, Poland

**Correction to: Digestive Diseases and Sciences** **https://doi.org/10.1007/s10620-025-09577-9**

Following publication, the authors identified an error in Table 2: Direct, indirect, and total effects of caregiver burden, health literacy, and Self-Efficacy on caregiver contribution to Self-Care domains (Maintenance, Monitoring, and Management) derived from structural equation modelling. During table preparation, some standardized estimates and corresponding 95% confidence intervals were inadvertently duplicated/misaligned across the three outcome domains. The underlying SEM analyses were not changed, and no re-analysis was performed; the correction is limited to replacing the published Table 2 with the corrected version reporting the appropriate domain-specific estimates. The corrected Table 2 corresponds exactly to the results described in the manuscript. This correction does not affect the scientific interpretation or conclusions of the article.

Correct version of Table 2:

**Table 2:** Direct, indirect, and total effects of caregiver burden, health literacy, and Self-Efficacy on caregiver contribution to Self-Care domains (Maintenance, Monitoring, and Management) derived from structural equation modelling

Effect	Caregiver contribution to Self-Care Maintenance	Caregiver contribution to Self-Care Monitoring	Caregiver contribution to Self-Care Management
	Estimate [95% IC]	Estimate [95% IC]	Estimate [95% IC]
Direct effects			
Caregiver burden → Caregiver contribution to Self-Care	**0.13 [0.01, 0.25]**	**0.30 [0.18, 0.42]**	**0.17 [0.03, 0.31]**
Caregiver self-efficacy → Caregiver contribution to Self-Care	0.11 [− 0.03, 0.23]	**0.34 [0.22, 0.46]**	**0.42 [0.29, 0.55]**
Health literacy → Caregiver contribution to Self-Care	− 0.01 [− 0.11, 0.07]	0.00 [− 0.14, 0.14]	− 0.01 [− 0.13, 0.10]
Age → Caregiver contribution to Self-Care	0.00 [− 0.01, 0.01]	0.00 [− 0.01, 0.01]	0.00 [− 0.01, 0.01]
Gender (male) → Caregiver contribution to Self-Care	0.03 [− 0.17, 0.22]	0.03 [− 0.17, 0.22]	− **0.19 [**− **0.33, **− **0.05]**
Employment (worker) → Caregiver contribution to Self-Care	− 0.14 [− 0.38, 0.09]	− 0.14 [− 0.38, 0.09]	− 0.14 [− 0.38, 0.09]
Pathology (UC vs CD) → Caregiver contribution to Self-Care	− **0.18 [**− **0.32,**− **0.04]**	− 0.05 [− 0.17, 0.04]	− 0.04 [− 0.12, 0.04]
Caregiver burden → Caregiver Self-Efficacy	− 0.13 [− 0.34, 0.07]	− 0.13 [− 0.34, 0.07]	− 0.13 [− 0.34, 0.07]
Health literacy → Caregiver Self-Efficacy	− 0.07 [− 0.17, 0.02]	− 0.07 [− 0.17, 0.02]	− 0.07 [− 0.17, 0.02]
Pathology (UC vs CD) → Caregiver Self-Efficacy	− 0.09 [− 0.28, 0.09]	− 0.09 [− 0.28, 0.09]	− 0.09 [− 0.28, 0.09]
Indirect effects			
Caregiver burden → Self-efficacy → Caregiver contribution	− 0.01 [− 0.05, 0.01]	− 0.01 [− 0.05, 0.01]	− 0.01 [− 0.05, 0.01]
Health literacy → Self-efficacy → Caregiver contribution	− 0.01 [− 0.03, 0.00]	− 0.01 [− 0.03, 0.00]	− 0.01 [− 0.03, 0.00]
Pathology (UC vs CD) → Self-efficacy → Caregiver contribution	− 0.01 [− 0.04, 0.01]	− 0.05 [− 0.17, 0.04]	− 0.04 [− 0.12, 0.04]

Bold values are statistically significant paths

*CD* Crohn's disease; *UC* ulcerative colitis, *SD* standard deviation, *SILS* Single Item Literacy Score, *ZBI* Zarit Inventory, *CSE-CSC* Caregiver Self-Efficacy in Contributing to Patient Self-Care Scale, *CC-SC-CII* Caregiver Contribution to Self-Care of Chronic Illness Inventory

