# Supplementary material for: Self-Efficacy as a Mediator Between Caregiver Burden, Health Literacy, and Contribution to Self-Care in Inflammatory Bowel Disease
Source: Dig Dis Sci. 2025 Dec 3;71(5):1731–7. doi: 10.1007/s10620-025-09577-9 (PMC13201276; doi:10.1007/s10620-025-09577-9)
Supplement: Supplementary file 2 — Supplementary file2 (DOCX 102 KB) [file 10620_2025_9577_MOESM2_ESM.docx]

**Supplementary File 2.** Descriptive subgroup distributions of caregiver contribution to self-care domains.

**
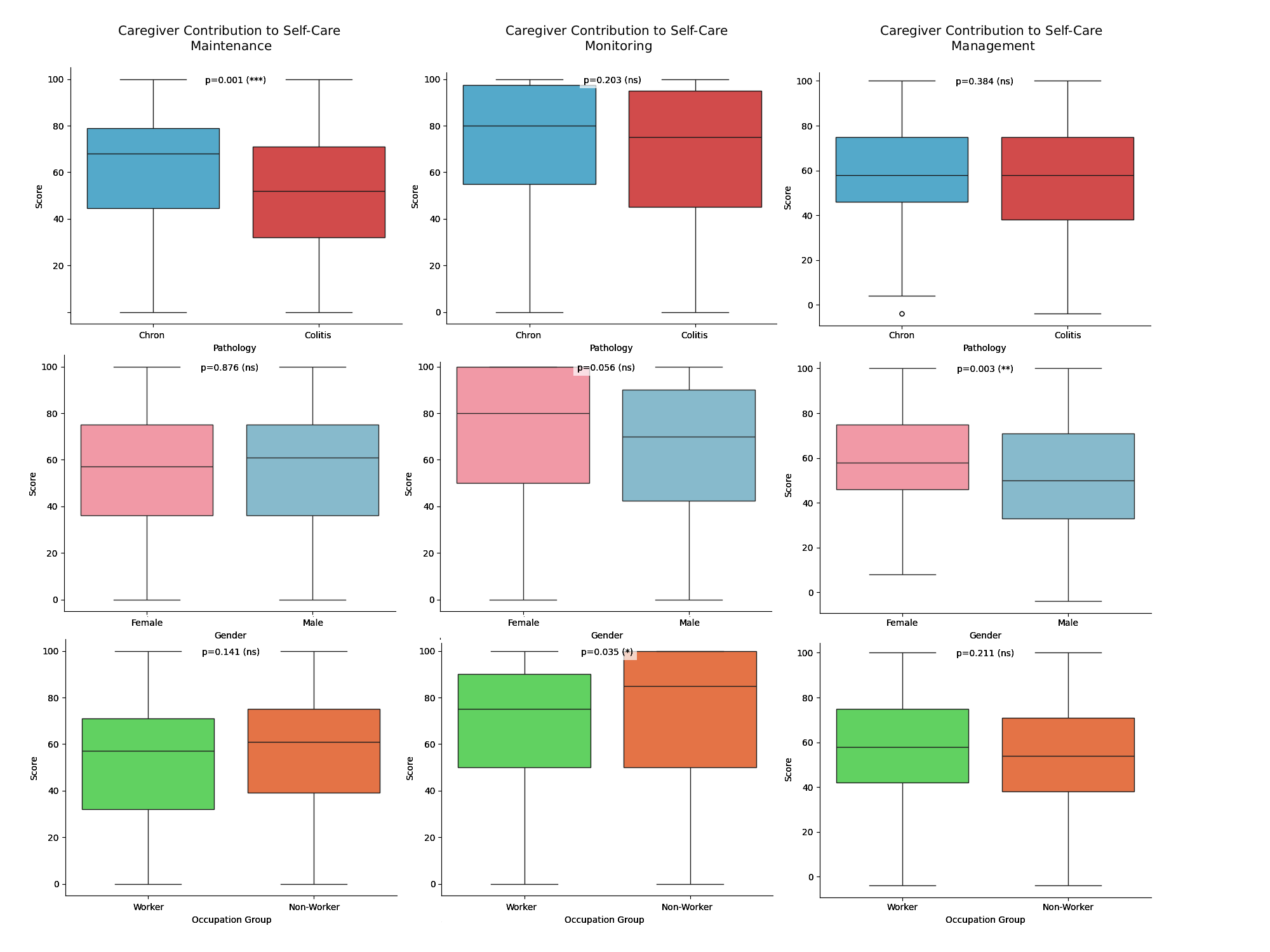
**

**Notes:** Boxplots show the unadjusted distributions of caregiver contribution to self-care (maintenance, monitoring, and management) across patient pathology, caregiver gender, and occupation group. These visualisations are descriptive only and are intended to provide a basic cohort picture. All inferential conclusions in this manuscript are drawn exclusively from the structural equation models.
